# Metagenomic next-generation sequencing assistance in identifying non-tuberculous mycobacterial infections

**DOI:** 10.3389/fcimb.2023.1253020

**Published:** 2023-08-31

**Authors:** Shilei Wang, Lihua Xing

**Affiliations:** Department of Respiratory and Critical Care Medicine, The First Affiliated Hospital of Zhengzhou University, Zhengzhou, China

**Keywords:** mNGS (metagenomic next-generation sequencing), NTM (nontuberculous mycobacteria), diagnosis, prognosis, culture

## Abstract

**Introduction:**

The advent of metagenomics next-generation sequencing (mNGS) has garnered attention as a novel method for detecting pathogenic infections, including Non-Tuberculous Mycobacterial (NTM) and tuberculosis (TB).However, the robustness and specificity of mNGS in NTM diagnostics have not been fully explored.

**Methods:**

In this retrospective study, we enrolled 27 patients with NTM genomic sequences via mNGS and conducted a comprehensive clinical evaluation.

**Results:**

Pulmonary NTM disease was the most commonly observed presentation, with a subset of patients also presenting with extrapulmonary NTM infections.mNGS analysis identified six distinct NTM species, primarily Mycobacteriumavium complex (MAC), followed by Mycobacterium intracellulare andMycobacterium abscessus. Conventional routine culture methods encountered challenges, resulting in negative results for all available 22 samples. Among the 10 patients who underwent quantitative polymerase chain reaction (qPCR) testing, five tested positive for NTM.

**Discussion:**

It is important to note that further species typing is necessary to determine the specific NTM type, as traditional pathogen detection methods serve as an initial step. In contrast, when supplemented with pathogen data, enables the identification of specific species, facilitating precise treatment decisions. In conclusion, mNGS demonstrates significant potential in aidingthe diagnosis of NTMdisease by rapidly detecting NTM pathogens and guiding treatment strategies. Its enhanced performance, faster turnaround time (TAT), and species identification capabilities make mNGS a promising tool for managing NTM infections.

## Introduction

*Non-tuberculous mycobacteria* (NTM), previously referred to as atypical mycobacteria, are a group of bacteria commonly found in the environment that can cause disease under specific conditions (Johansen et al., 2020). NTM encompasses mycobacterial species other than Mycobacterium tuberculosis, *Mycobacterium bovis*, and *Mycobacterium leprae*. While sharing some antigenic characteristics with *M. tuberculosis*, NTM exhibit distinct properties, such as increased susceptibility to pH changes (acids and bases)(Gopalaswamy et al., 2020). Additionally, NTM display greater tolerance to commonly used anti-tuberculosis drugs and can grow at temperatures less stringent than those required by *M.tuberculosis*, potentially leading to tuberculosis-like lesions (Cowman et al., 2019; Ratnatunga et al., 2020).

Pulmonary NTM infections account for 90% of all NTM cases, emphasizing the importance of focusing on pulmonary NTM infections (Falkinham, 2016; Wassilew et al., 2016). While NTM infections primarily affect the lungs and cause bacillary pneumonia, they can also occur in other parts of the body. Symptoms and signs of NTM pulmonary disease (NTM-PD) are nonspecific. CT examinations typically reveal various manifestations in the lungs, including bronchiectasis, nodules, cavities, and solid lesions, often presenting with multiple CT findings (Gopalaswamy et al., 2020; Kumar and Loebinger, 2022; Pathak et al., 2022; Varley and Winthrop, 2022).

Conventional microbial culture methods used for diagnosing NTM infections present challenges due to their long culture time and low positive culture rate. To overcome these limitations, metagenomic next-generation sequencing (mNGS) technology has emerged as a promising DNA/RNA sequencing method. mNGS offers higher throughput by amplifying all nucleic acid sequences in the sample with the potential to detect all possible pathogens without bias (Han et al., 2019; Diao et al., 2022). This unbiased approach is particularly valuable for the early diagnosis of difficult-to-detect infections. Consequently, mNGS is increasingly being employed in clinical practice, playing a significant role in improving diagnostic capabilities (Li et al., 2021; Gökdemir et al., 2022). However, the diagnostic potential and specificity of mNGS in NTM diagnosis are still being evaluated in clinical practice. While mNGS has shown promise as a novel method for detecting respiratory pathogenic infections, including NTM, further research and validation studies are needed to assess its specificity and reliability compared to traditional diagnostic methods.

In this retrospective study, we acknowledge the need for further species typing and the importance of combining mNGS with other pathogen detection methods as an initial step. This approach allows for the identification of specific NTM species, which is crucial for guiding precise treatment decisions. While mNGS can rapidly detect NTM pathogens and provide species identification, it should be supplemented with other diagnostic tools to confirm and validate the findings. We aimed to assess the diagnostic potential of mNGS in 27 patients with NTM genomic sequences identified at our hospital from March 2021 to March 2023. Our objective was to evaluate the effectiveness and accuracy of mNGS as a diagnostic tool for NTM by integrating the patients’ clinical characteristics. We comprehensively analyzed the patients’ clinical data, including symptoms, medical history, and imaging findings, in conjunction with mNGS results to determine the performance of mNGS in diagnosing NTM infections.

## Materials and methods

### Patient recruitment

A retrospective study was conducted at the First Affiliated Hospital of Zhengzhou University to evaluate the diagnostic potential of metagenomic next-generation sequencing (mNGS) for *non-tuberculous mycobacterial* (NTM) infections. Patients with NTM detected in clinical specimens using mNGS between March 2021 and March 2022 were included in the study. Diagnosis of NTM infection was made by clinical experts based on diagnostic criteria, including the Guidelines for the diagnosis and treatment of non-tuberculous mycobacterial disease (2020 edition), clinical presentation, laboratory examination, microbiological examination, and radiological examination. A total of 27 patients who met the entry criteria were enrolled in the study. Demographic characteristics, clinical history, laboratory tests, mNGS results, polymerase chain reaction (PCR) results, and microbiological culture information were collected and recorded in the hospital information system.

### Specimen collection

Four types of specimens were collected, including 21 bronchoalveolar lavage fluid (BALF) samples, 4 lung tissue specimens, 1 abscess specimen, and 1 pleural fluid specimen. BALF, pleural fluid, and abscess samples were collected in quantities of 5ml or more and stored in 50ml cryopreservation tubes. Lung tissue samples larger than 3 x 3 x 3 mm³ were placed in 5 ml dry ice cryopreservation tubes for transport.

### Diagnostic process efficiency

To assess the efficiency of the diagnostic process, several time points were defined. The sample time was the duration from the date of admission to the date of sample collection. Turnaround time (TAT) referred to the period from sample submission for mNGS or microbial culture to the receipt of the report. Result time was calculated as the time elapsed from the date of admission to the receipt of the mNGS or microbial culture report.

### Metagenomics NGS and bioinformatics

Samples were processed using the NGSmaster™ (Hangzhou Jieyi) automated workstation for nucleic acid extraction, reverse transcription (for RNA samples), nucleic acid fragmentation, end-complementation, terminal adenylation, sequencing adapter ligation, and purification, resulting in the formation of sequencing libraries. Library quantification was performed using fluorescent quantitative PCR, and the libraries were subsequently sequenced using the Illumina Nextseq™ 550 high-throughput sequencing platform with a targeted output of 20 million 50 bp single-end sequence data per read.

Bioinformatic analysis was conducted to analyze the library sequence data. Human genomic sequence data were filtered out, and the remaining sequence data were compared to a microbial reference database to determine microbial species, sequence number, and relative abundance.

Quality control measures included the inclusion of negative controls (dummy samples containing human-derived nucleic acids) and positive controls (inactivated pseudovirus particles) to monitor background microorganisms and RNA sequencing quality.

Reporting rules for mNGS analysis included meeting quality control requirements for sequence data and discrimination between background and true microorganisms using negative control analysis.

The mNGS reporting rules are: 1) the sequence data meet the quality control requirements (library concentration > 50 pM, Q20 > 85%, Q30 > 80%); 2) the negative control (NC) does not contain the species detected in the same microarray or has an RPM (sample)/RPM (NC) ≥ 5, thus discriminating between background and true microorganisms.

### Routine culture

Pre-treatment of sputum specimen culture: add equal volume of diluted sputum digest to the sputum, vortex the specimen for 20-30 seconds and leave it at room temperature for 15 minutes; vortex again to further liquefy the specimen, and prepare it for immediate inoculation of the specimen. Take the suspension with a swab into the culture medium and draw a line.

General bacterial culture: inoculate the treated specimen on Columbia blood plate and chocolate plate, put it into 35°C, CO2 incubator environment, observe the characteristics of colonies and smear for gram staining after 18-24 hours, and make preliminary identification according to the morphology of the organism. According to the morphology and characteristics of the organism, the preliminary identification will be made.

Mycobacterium tuberculosis culture: before the first sputum inoculation of stray bacteria in the modified Roche medium, placed in 35°C culture, generally the first week to observe the peal times, and then once a week to observe the time of colony appearance, morphology, positive results at any time. Morphology, positive results at any time to send a report, the negative 6 weeks later to send a report. (4) Fungi with sand Paul plate and Komaja fungi color plate culture, after inoculation put 28 °C ordinary incubator.

### Statistical analysis

Statistical analyses were performed using R version 3.6.1. Categorical variables were presented as counts and percentages and compared using Fisher’s exact test. Continuous variables were expressed as means with standard deviations (SD) and compared between groups using the Wilcoxon rank sum test. Statistical significance was defined as a two-tailed p-value less than 0.05.

## Results

### Patients’ characteristics

1. Baseline information and clinical characteristics of all patients are summarized in **Table 1**.
  Among the 27 patients included in the study, 9 were male, and 18 were female. The age range of the patients varied from 30 to 79 years, with a mean age of 58.41 years. 14 patients were aged over 60 years, comprising approximately 52% of the total patient cohort. Regarding hospitalization details, only one patient among the 27 had been admitted to the intensive care unit and was on a ventilator for an extensive duration of 2787 hours. The remaining patients did not require intensive care or ventilation. In terms of the length of hospital stay, most of the NTM patients had relatively shorter stays of less than 20 days. However, four patients experienced a longer length of stay, exceeding 20 days. Clinical symptoms reported by the patients revealed that cough was the most prevalent symptom, followed by fever, chest tightness, chest pain, and hemoptysis. Additionally, 17 out of the 27 patients had one or more underlying medical conditions, including chronic lung diseases such as bronchiectasis and COPD, post-surgical conditions (lung tumor, lung transplant, and gynecological tumor), and other medical conditions such as hypertension, diabetes mellitus, and coronary heart disease. Regarding the type of NTM infection, 25 patients were diagnosed with intrapulmonary NTM infection, while the remaining 2 patients had extrapulmonary NTM infection.

**Table 1:** Patients’ characteristics.

Characteristics	Total (n=27)
**Age, mean ± sd**	58.41 ± 13.23
Gender, no. (%)	
Male	9(33.33)
Female	18(66.67)
**ICU, no. (%)**	1(3.70)
ICU days	109 ± 0
**Ventilator, no. (%)**	1(3.70)
Ventilator hours	2787 ± 0
Hospital days, no. (%)	
<10 days	12(44.44)
10-30days	11(40.75)
>30days	4(14.81)
Main symptoms, no. (%)	
Fever	8(29.6)
Cough	11(40.74)
Chest tightness	3(11.11)
Chest pain Hemoptysis	2(7.41) 3(11.11)
Dyspnea	1(3.70)
Local Dermal rupture Coma	1(3.70) 1(3.70)
Underlying diseases, no. (%)	
Hypertension	3(11.11)
Diabetes	4(14.81)
Bronchiectasis COPD	6(22.22) 2(7.41)
Connective tissue disease	1(3.70)
Heart diseases Hyperlipidemia Cerebral infarction High paraplegia	2(7.41) 1(3.70) 1(3.70) 1(3.70)
Lung cancer	2(7.41)
After gynecological tumor surgery Post-lung transplantation	3(11.11) 2(7.41)
*NTM* infection sites, no. (%)	
Pulmonary	25(92.59)
Extrapulmonary	2(7.41)

ICU, intensive care unit; COPD, Chronic obstructive pneumonia.

2. The mNGS and microbial culture information is summarized in **Table 2**.
  Among the 27 patients included in the study, samples were collected from four different types: 21 patients provided bronchoalveolar lavage fluid (BALF), three patients provided lung tissue samples, one patient provided thyroid tissue, one patient provided pleural effusion, and one patient provided an abscess sample. Microbiological cultures were conducted in 22 of these 27 patients, but all of the cultures yielded negative results. The turnaround time (TAT) for mNGS was only 1 day in 23 patients, while only 1 out of the 22 cultures had a TAT of 1 day. This indicates that the average TAT for mNGS was significantly shorter compared to culture-based methods (1.19 days vs. 3.86 days, respectively, p<0.001).
  The mean time to receive the mNGS result from the time of admission to the hospital was 14.19 days, while the mean time for the culture result was 8.14 days. However, there was no statistically significant association between the two (p>0.05). It is worth mentioning that among the 27 patients, a few individuals required admission to the intensive care unit and ventilator support for an extended period due to severe underlying diseases. Consequently, their time to receive mNGS and culture results was significantly prolonged, impacting the overall statistics of mNGS and culture result times.

**Table 2:** mNGS and microbiological culture information.

Characteristics	Total(n=27)	P value
Specimen types, no. (%)		
BALF	21(55.56)	
Lung tissue	3(11.11)	
Thyroid tissue Pleural effusion	1(5.56) 1(5.56)	
Abscess	1(5.56)	
**Specimen time, mean ± sd**	8.11± 11.97	
**mNGS turnaround time day, mean ± sd**	1.19 ± 0.48	<0.001
**Culture turnaround time day, mean ± sd**	3.86 ± 2.10	
**mNGS result time day, mean ± sd**	14.19 ± 20.33	0.71
**Culture result time day, mean ± sd**	8.14 ± 6.50

3. The imaging characteristics of the patients are summarized in **Table 3**.
  We collected imaging data from a total of 27 patients, with CT scans available for all except two patients who had their CT scans performed outside the hospital. Analysis of the CT scans revealed several common patterns of NTM infection distribution. Most patients exhibited lesions in the upper regions of both lungs, near the pleura. However, in some patients, the infection was observed in the lower lung regions, while in others, the infection involved the hilar lymph nodes. Lung nodules were detected in 18 out of 25 patients (72%), and 20 patients (80%) displayed patchy or solid lung shadows. Approximately 30% of the patients exhibited various imaging findings, including ground-glass opacities, fibrous ground-glass opacities, bronchiectasis, and the tree-bud sign. Additionally, a few individual patients displayed positive pleural cavities, pleural thickening, and emphysema. Notably, none of the 25 patients showed signs of pulmonary atelectasis.

**Table 3:** Patient’s imaging characteristics.

case.No		nodule	plaque1/small plaque/solid shadow, 2	cavitation	Dilated bronchial tubes	tree bud sign	ground glass shadow	fibrous streak shadow	pulmonary emphysema	pulmonary atelectasis	pleural effusion	pleural thickening
1	Double lower lung, proximal pleura	0	1	0	0	0	0	1	0	0	0	0
2	Both upper lungs, proximal pleura	0	2	0	0	0	1	0	0	0	1	0
3	Right lung	1	2	1	1	0	1	1	1	0	0	0
4	center lung	1	1	0	0	0	1	1	0	0	1	0
5	NA	NA	NA	NA	NA	NA	NA	NA	NA	NA	NA	NA
6	Right upper lung	0	1	0	1	1	0	0	0	0	0	0
7	Double upper lung	1	1	1	1	0	0	1	0	0	0	0
8	right upper lung	1	1	0	0	0	0	0	0	0	0	0
9	right lower middle lung	1	2	0	0	0	1	0	0	0	0	0
10	Right lung	1	0	0	0	1	0	1	0	0	0	0
11	Double upper lung	1	1	1	1	0	0	0	0	0	0	0
12	right lung	0	2	1	0	0	0	1	0	0	0	0
13	Right middle lung	1	1	0	0	0	0	0	0	0	0	0
14	center lung	1	0	1	0	0	0	1	0	0	0	1
15	Both lungs, near the pleura	1	1	0	0	0	1	1	0	0	0	0
16	center lower lung	0	1	0	0	0	1	0	0	0	0	0
17	center lower lung (granulomatous inflammation of mediastinal lymph nodes)	0	0	0	0	0	0	1	0	0	0	0
18	Both lungs	1	1	1	1	0	0	0	0	0	0	0
19	Right lower lung	0	0	0	0	0	0	1	0	0	0	1
20	Middle lobe of right lung	1	0	0	0	1	0	0	0	0	0	0
21	Double upper lung	1	1	0	0	0	0	0	0	0	0	0
22	Double upper lung, near pleura	1	1	0	0	0	0	0	0	0	0	0
23	Upper lobe of right lung	1	1	0	0	0	0	0	0	0	0	0
24	Both lower lungs	1	2	1	0	0	1	0	0	0	0	0
25	center lung lingual lobe	1	1	0	1	1	0	0	0	0	0	0
26	Upper lobe of right lung	1	1	0	0	0	0	0	0	0	0	0
27	NA	NA	NA	NA	NA	NA	NA	NA	NA	NA	NA	NA

### Results for mNGS

**Figure 1:**
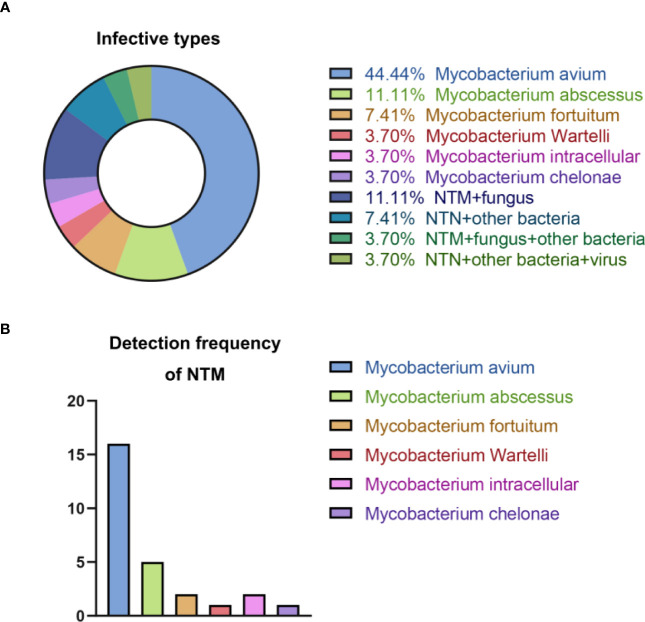
Summary of NTM infections. **(A)** Types of NTM infection; **(B)** Detection frequency of NTM.

1. All 27 patients had NTM infections detected by mNGS and the type of NTM infection is shown in **Figure 1A**.
  Among the 27 patients included in the study, 74% had a single NTM infection. The most prevalent single NTM infection was *Mycobacterium avium complex* (MAC), accounting for 44% of all infections. Other single NTM infections included *Mycobacterium abscessus* (11%), *Mycobacterium occasionalis* (7%), *Mycobacterium wolframei* (4%), *Mycobacterium intracellulare* (4%), and *Mycobacterium torulare* (4%).
  Regarding mixed NTM infections, which involve one or more NTMs along with other bacteria, fungi, or viruses, all the mixed infections observed in the 27 patients consisted of one type of NTM along with other microorganisms. The most common mixed infections were NTM + fungus, accounting for 11% of all infections. This was followed by NTM + other bacteria (7%), NTM + fungus + other bacteria (4%), and NTM + other bacteria + virus (4%).
  In our analysis of mNGS results from the 27 patients, we detected a total of six different types of NTM. *Mycobacterium avium complex* (MAC) was the most frequently detected, occurring 16 times. *Mycobacterium abscessus* was detected five times, while *Mycobacterium avium* and *Mycobacterium intracellulare* were each detected twice. *Mycobacterium turtle* and *Mycobacterium wolfram* were detected once each. These findings suggest that MAC is likely the most common type of NTM infection detected in our hospital (**Figure 1B**).

2. A comparison of mNGS, PCR, and conventional culture results is presented in **Table 4**.
  Among the 27 patients included in the study, conventional culture was performed on samples from 22 patients. However, all of these culture results were negative. 10 patients also underwent qPCR testing using a universal NTM primer, 5 tested positive for NTM infection, indicating the presence of NTM DNA in their samples.

**Table 4:** Comparison of mNGS, PCR, and conventional culture.

	mNGS testing results	Conventional culture results	Pcr testing results
Positive	27	0	5
Negative	0	27	5

### Prognosis of patients

During the follow-up period, we monitored the prognosis of the 27 patients who participated in the study. Out of the 26 surviving patients, one patient unfortunately passed away due to severe underlying diseases, specifically paraplegia and respiratory and circulatory failure. For the remaining patients, we collected data on their follow-up CT scans to assess the progression of the disease. The follow-up period varied from half a month to nine months, with the majority of patients having follow-up scans between one and three months after the initial assessment. Among the patients with available follow-up CT data (12 patients), we compared the pre- and post-treatment CT findings.

**Figure 2:**
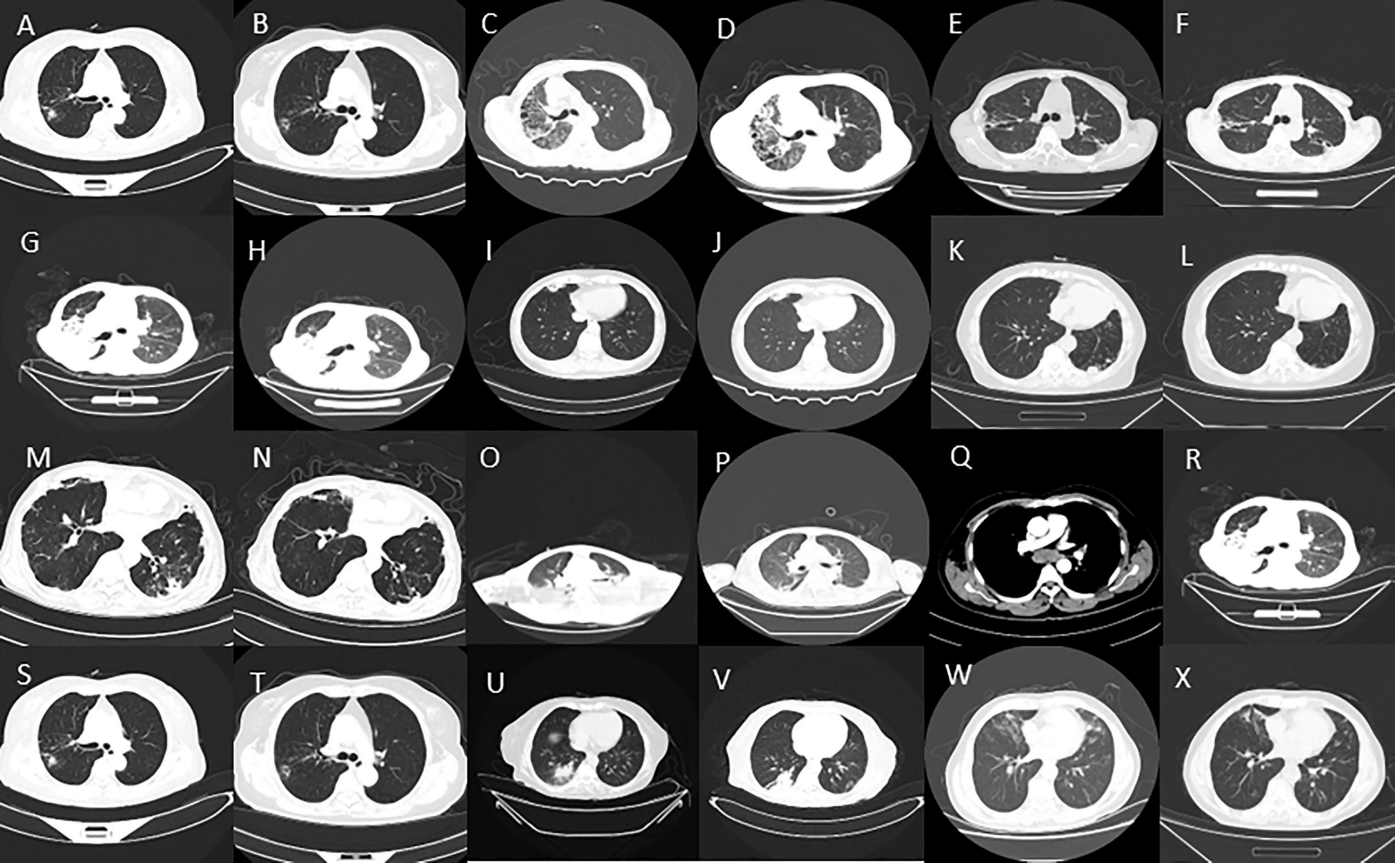
**(A–X)** Imaging features of some NTM patients before and after treatment.

Out of these 12 patients, nine showed either complete resolution or apparent improvement of lung lesions in their follow-up CT scans. This indicates a positive response to treatment. On the other hand, three patients did not show any resolution of their lung lesions in the follow-up CT scans. These patients had their follow-up CT scans at different time intervals: half a month, three months, and six months, respectively. Upon reviewing the medical records, we found that five out of the 12 patients underwent changes in their medication regimen, including the administration of specific antibiotics, after the detection of NTM infection by mNGS. This suggests that tailored treatment interventions based on mNGS results were implemented for these patients (**Figure 2**).

## Discussion

In this retrospective study, our primary objective was to assess the utility and specificity of metagenomic next-generation sequencing (mNGS) in diagnosing and managing non-tuberculous mycobacterial (NTM) disease. We enrolled a total of 27 patients based on the detection of NTM genomic sequences through mNGS. Additionally, we thoroughly evaluated the patients’ clinical characteristics, underlying diseases, site of infection, treatment history, and prognosis, along with other laboratory tests including qPCR. All 27 patients included in the study were diagnosed with NTM infection based on a combination of clinical presentation and examination results, as determined by specialists. This comprehensive approach ensured a reliable diagnosis and enhanced the accuracy of our findings. Consistent with previous studies, we found that mNGS had a faster turnaround time (TAT) compared to microbial culture. Moreover, the positive predictive value of mNGS was higher than that of culture, aligning with existing studies (Miao et al., 2018; Chen et al., 2021). Another key advantage of mNGS over PCR assays is its ability to provide specific typing of NTM strains, enabling clinicians to make well-informed medication decisions more rapidly.

*Non-tuberculous mycobacteria* (NTM) are mycobacterial species excluding *Mycobacterium avium complex* (MAC), *Mycobacterium leprae*, and *Mycobacterium abscessus complex* (MABC) as the primary types (Haworth et al., 2017; Daley et al., 2020) The incidence of NTM infections has been gradually increasing globally, with Asian populations experiencing a rising incidence rate of approximately 39.6 cases per 100,000 person-years, increasing at a rate of 19 cases per 100,000 person-years annually (Shteinberg et al., 2018). In China, as tuberculosis control measures have improved, the incidence of NTM infections has also been gradually increasing. Clinical data from Miao et al. (2018) indicates that approximately 47% of patients with infectious diseases are infected with Mycobacterium avium, of which approximately 50% are attributed to NTM. However, it is worth noting that the detection rate of NTM is lower than its actual incidence due to limitations in diagnostic techniques (Raju et al., 2016). Addressing the challenges associated with the diagnosis of NTM infections is crucial for accurate identification and appropriate management. Advancements in diagnostic techniques, such as mNGS, hold promise in enhancing the detection and typing of NTM, enabling more effective diagnosis and treatment.

The distribution of *non-tuberculous mycobacteria* (NTM) in nature is influenced by various factors, including geographical location and climate, resulting in variations in the incidence of NTM disease across different regions (Sharma and Upadhyay, 2020). In our institution, the most commonly detected NTM species among NTM infections was the *Mycobacterium avium complex.* Out of the 27 patients with NTM infections in this study, *Mycobacterium avium nontuberculosis complex* was found in 16 patients, followed by *Mycobacterium abscessus*, *Mycobacterium occasionalis*, and *Mycobacterium intracellulare*. However, it is important to note that the predominant NTM species may vary by region. For example, in Beijing, China, M*ycobacterium intracellulare* and *Mycobacterium kansasii* were the most common, while in Shanghai, *Mycobacterium kansasii* was the most prevalent, followed by *Mycobacterium intracellulare* and *Mycobacterium tortugae/abscessus*.

The pathogenesis of *non-tuberculous mycobacterial* (NTM) infections is characterized by its occurrence at any age, with a higher prevalence in women and the elderly, particularly menopausal women. Several underlying lung diseases, such as tuberculosis, bronchiectasis, chronic obstructive pulmonary disease (COPD), cystic fibrosis, pneumoconiosis, primary ciliary dyskinesia, alpha1-antitrypsin deficiency, allergic bronchopulmonary aspergillosis, chest deformities, chest tumors, and post-lung transplantation, have been identified as important risk factors for NTM infection (Billinger et al., 2009; Cassidy et al., 2009; Prevots et al., 2010; Ratnatunga et al., 2020).

In our study, out of the 27 patients included, 20 were female and 13 were over 50 years of age. Furthermore, 18 of the 27 patients had varying degrees of underlying diseases, such as post-operative lung tumors, post-lung transplantation, post-operative gynecological tumors, and chronic obstructive pulmonary disease. The clinical manifestations of NTM infection are similar to those of tuberculosis, including systemic toxicity and local damage, but the symptoms of systemic toxicity are generally less severe than those of tuberculosis. Patients with NTM infection may present with a range of symptoms, from no obvious symptoms for an extended period to cough and sputum production. Some patients may experience rapid disease progression with symptoms such as cough, sputum production, hemoptysis, chest pain, chest tightness, shortness of breath, night sweats, low-grade fever, weakness, and wasting (Gopalaswamy et al., 2020).

The diagnosis of *non-tuberculous mycobacterial* (NTM) infections can be challenging, and the guidelines mentioned highlight the importance of NTM cultures and molecular biological testing in the diagnostic process (Pennington et al., 2021). However, it is noted that the positive rate of microbiological cultures for NTM can be low, and the culture process itself can be time-consuming and difficult, especially considering the growth requirements of some NTM strains. Additionally, there is currently no widely available technique for directly identifying NTM species from clinical specimens. While culture remains the primary step for identification, further diagnostic methods are necessary to determine the species (Pennington et al., 2021). IOngoing research aims to develop methods for the direct identification of NTM organisms from clinical samples. Polymerase chain reaction (PCR)-based methods are being explored for rapid identification of NTM species in respiratory samples, but their accuracy for strain typing is still limited (Kim and Park, 2019). In our study, none of the 27 patients had positive results in microbiological cultures. This underscores the challenges associated with culture-based identification of NTM. However, qPCR testing was performed in 10 patients, with five showing positive results. Further identification of the specific infecting NTM species is necessary for these patients. Continued advancements in diagnostic techniques for NTM infections are important to improve the accuracy and efficiency of identification, which, in turn, will aid in appropriate management and treatment decisions.

mNGS is an innovative technology increasingly utilized for the detection of infectious agents. It represents a breakthrough in pathogen detection due to its use of random primers for unbiased amplification and its higher sensitivity compared to traditional detection methods (Street et al., 2017; Chiu and Miller, 2019; Lee et al., 2019; Miller et al., 2019). One of the key advantages of mNGS is its high sensitivity, enabled by the ability to sequence DNA at a sufficient depth, allowing for the accurate detection of pathogens even when they are present in low numbers. Additionally, mNGS has the capability to simultaneously and independently sequence large amounts of DNA, without prior knowledge of the specific pathogen. This is particularly advantageous in the context of NTM infections, as a significant proportion of patients with NTM infections often have underlying lung diseases such as bronchiectasis and chronic obstructive pulmonary disease (COPD). These patients may harbor multiple infections that are challenging to detect using conventional culture or molecular techniques targeting a single pathogen. Consequently, misdiagnosis or missed diagnoses can occur (David, 1987). In the case of NTM infections, sputum is the most accessible specimen for repeated testing. However, invasive procedures like bronchoscopy or tissue puncture to obtain specimens can be difficult for some patients. Therefore, the development of a method that can detect pathogens from a single specimen would be highly beneficial, as it would provide valuable guidance for clinical diagnosis and treatment.

Our study findings highlight the advantages of mNGS in diagnosing NTM infections with co-infections by other pathogens, as well as its shorter detection time compared to traditional culture-based methods. The ability of mNGS to detect co-infections involving *Pseudomonas aeruginosa*, *Aspergillus*, and viruses demonstrates its superiority over other molecular detection techniques in identifying multiple pathogens concurrently. The rapid turnaround time of 2-3 days for obtaining pathogenic evidence with mNGS is particularly advantageous compared to the average feedback time of 7-14 days for culture-based methods. This expedited diagnosis supports the recognition of mNGS as a valuable tool for early and rapid disease diagnosis in clinical practice (Aoki and Kushimoto, 1987; Swenson et al., 2018; Han, 2022). Additionally, our study highlights another advantage of mNGS, which is its ability to directly target specific NTM strains without the need for further strain typing. This feature saves time and cost while enabling clinicians to guide precise dosing decisions for NTM infections (Gopalaswamy et al., 2020) (Gökdemir et al., 2022). By providing information on the specific NTM strain, mNGS facilitates tailored antibiotic treatment, which can lead to improved patient outcomes. Medication adjustments based on mNGS results led to improved prognosis for five patients during follow-up.

It’s also important to acknowledge the limitations of our study, including the small sample size of 27 patients. The limited prevalence of NTM may have contributed to the small sample size. Additionally, as mNGS is a relatively new technology in our institution, the retrospective nature of this single-center study further restricts the sample size. It’s crucial to validate the diagnostic efficacy of mNGS for NTM in larger sample sizes to establish more reliable conclusions. (Nayak, 2010). Furthermore, the complexity of NTM cultures prevented the screening of patients with positive NTM cultures, which is another limitation to consider.

## Conclusion

In our study, the clinical characteristics and infection status of patients with clinically diagnosed NTM disease who tested positive for mNGS were evaluated. The turnaround time (TAT) of mNGS was found to be faster compared to microbial cultures, which is beneficial in the timely diagnosis of NTM infections. The complexity of NTM culture resulted in all NTM cultures being negative, underscoring the challenges associated with conventional culture-based methods for NTM detection.

An important advantage of mNGS is its ability to directly type NTM infections without the need for further strain identification, which is required with culture or PCR-based methods. This direct typing enables immediate initiation of targeted treatment based on the identified pathogen type. This aspect of mNGS facilitates the precise clinical use of drugs and optimizes the treatment process for NTM infections.

Overall, our study suggests that mNGS can rapidly and accurately identify NTM pathogens, leading to improved clinical management by guiding precise drug selection. The advantages of mNGS, including its faster TAT and direct typing capabilities, make it a valuable tool in the diagnosis and treatment of NTM infections.

## Data availability statement

The datasets presented in this study can be found in online repositories. The names of the repository/repositories and accession number(s) can be found below: EMBL with accession number PRJEB61771.

## Ethics statement

The study was approved by the institutional review board of the First Affiliated Hospital of Zhengzhou University. The ethics approval number is 2020-KY-142. The studies were conducted in accordance with the local legislation and institutional requirements. Written informed consent for participation was not required from the participants or the participants’ legal guardians/next of kin in accordance with the national legislation and institutional requirements.

## Author contributions

SW analyzed patients’ data. SW participated in the writing of the manuscript. LX designed the study and conceived the project. All authors read and approved the final manuscript.
