# Supplementary material for: Metagenomic next-generation sequencing assistance in identifying non-tuberculous mycobacterial infections
Source: Front Cell Infect Microbiol. 2023 Aug 31;13:1253020. doi: 10.3389/fcimb.2023.1253020 (PMC10500063; doi:10.3389/fcimb.2023.1253020)

Kouwanshun

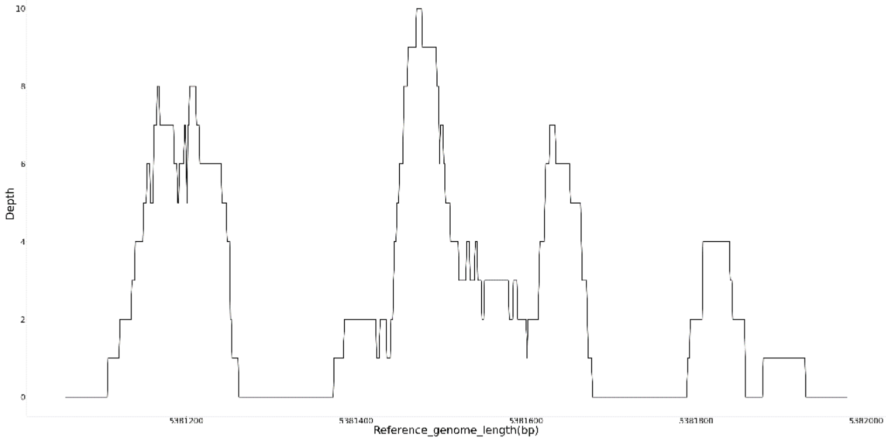

Zhangbaoping

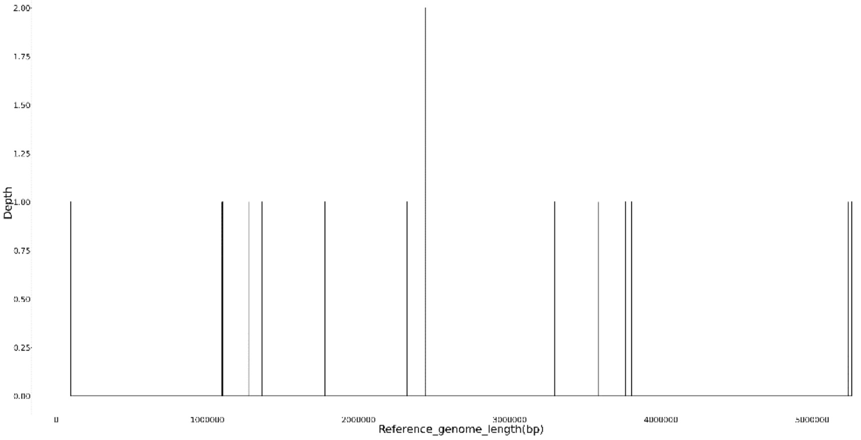

Yangwenfa

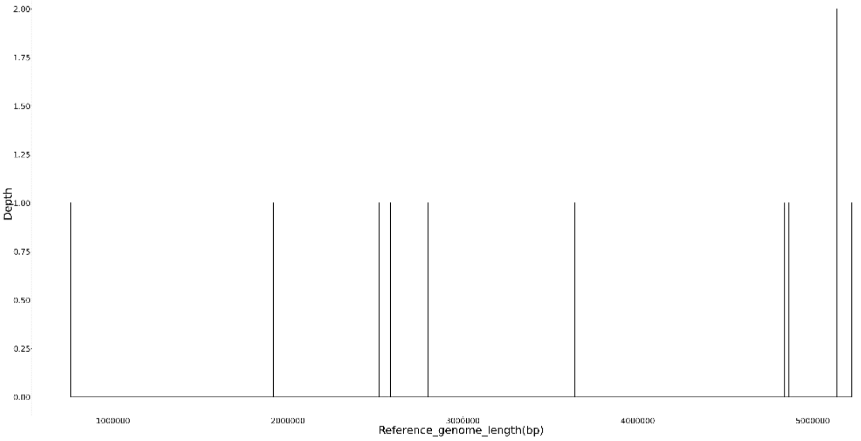

Wangkui

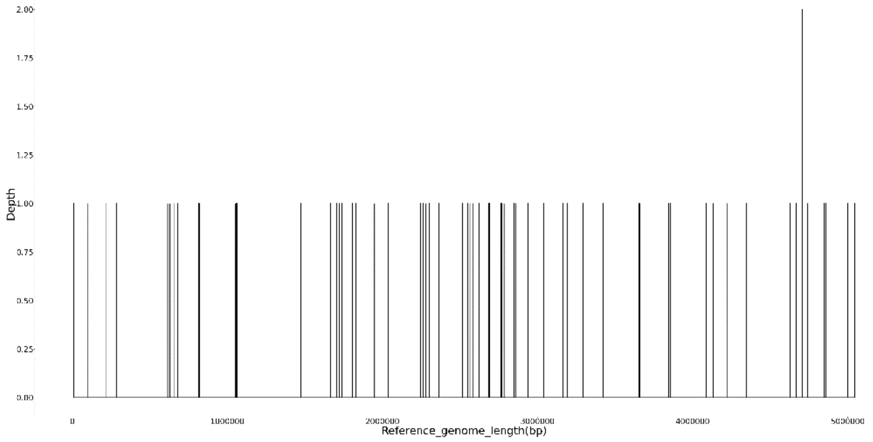

Bihexiang

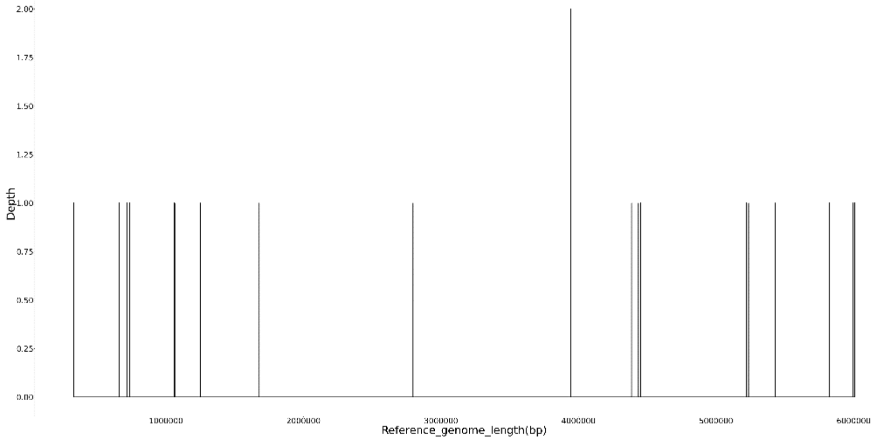

Jiafurong

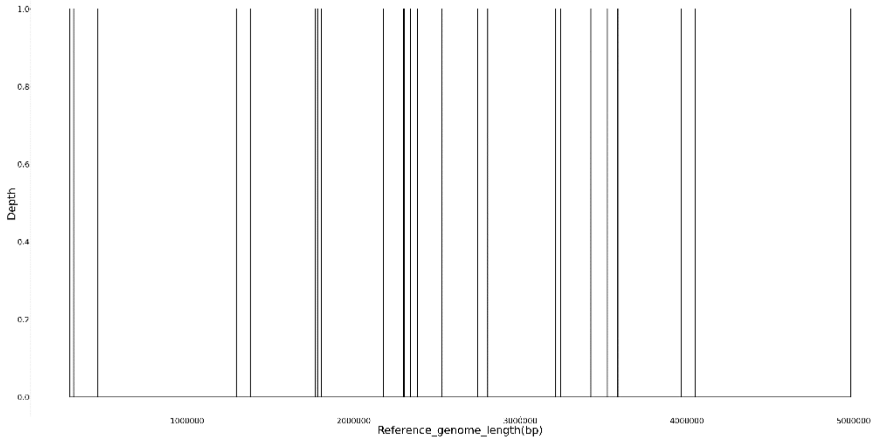

Jingzhohua

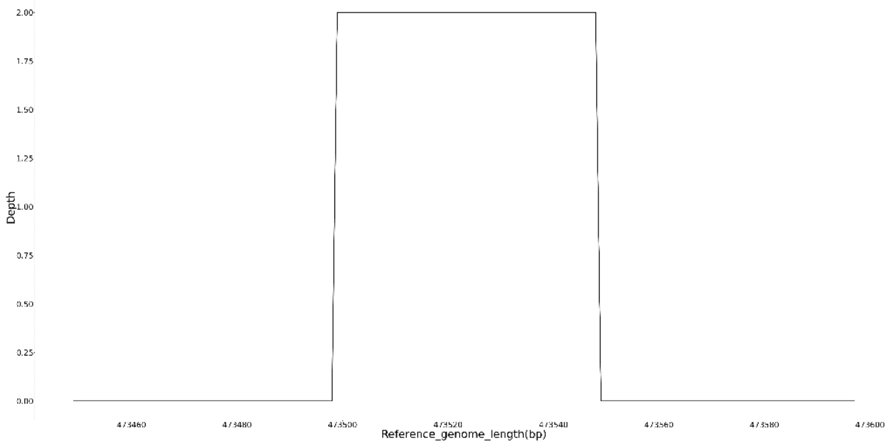

Lisuzhi

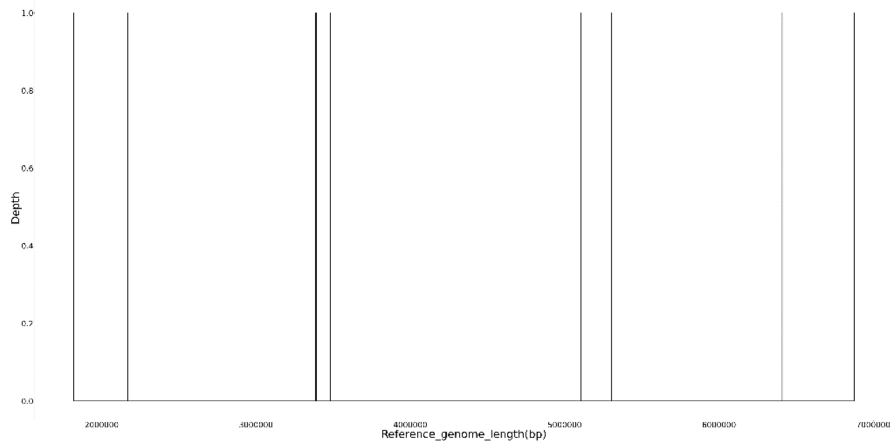

Liucunying

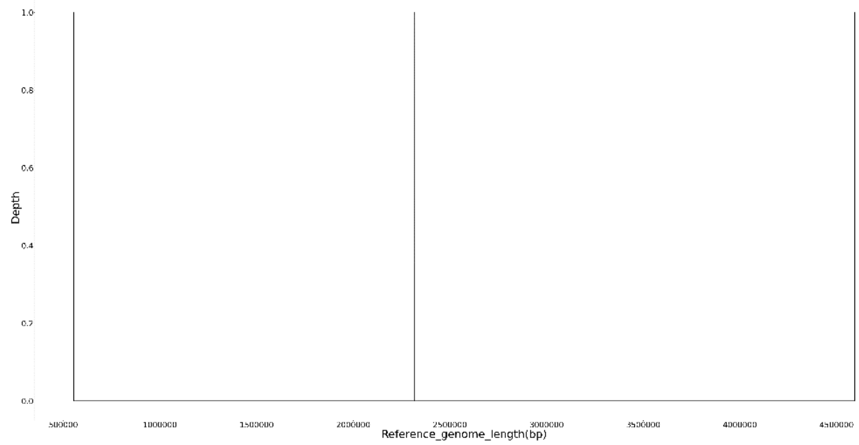

Liuhziwe

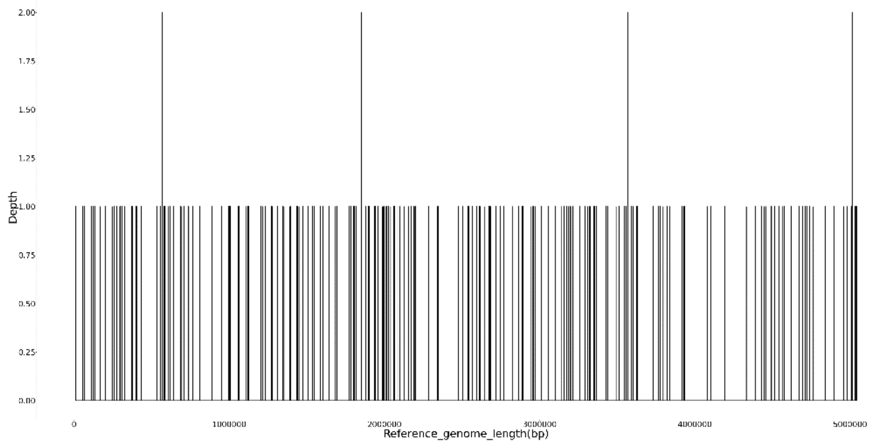

Shangzhence

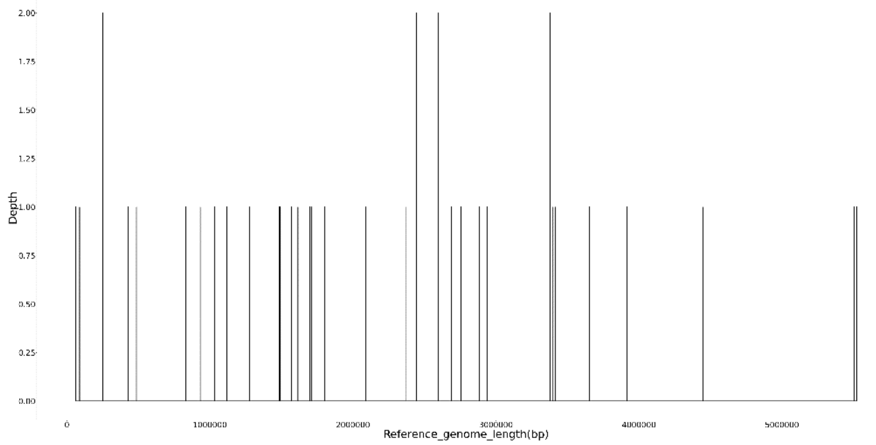

Shenghengying

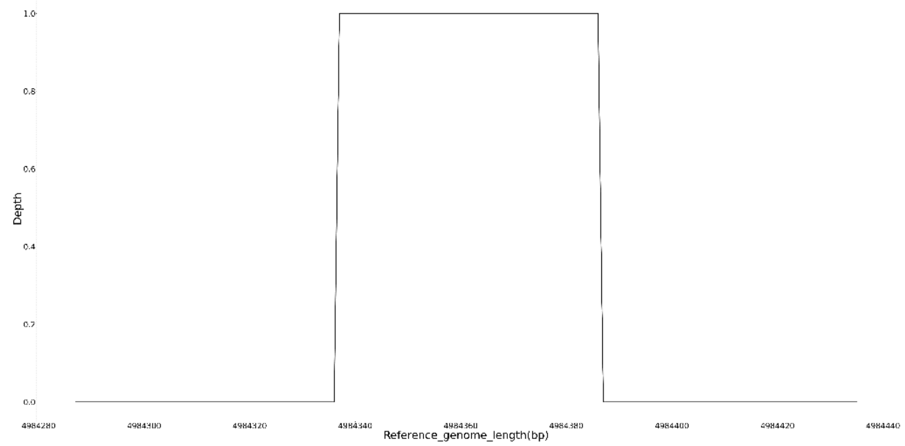

Wangjinming

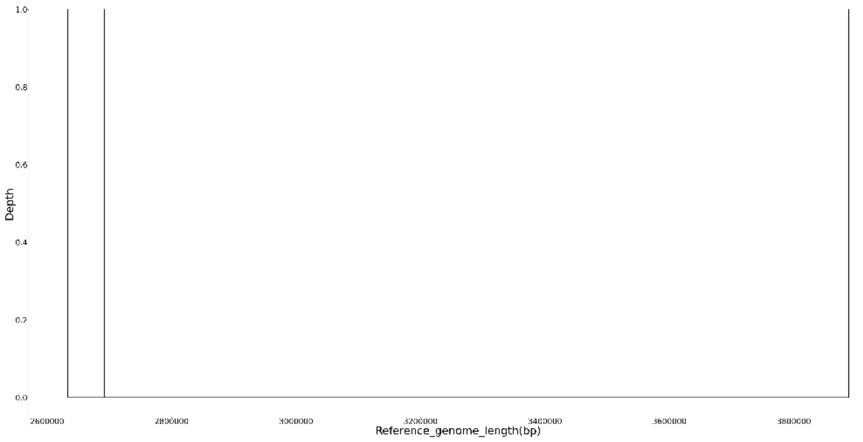

Weiyanxia

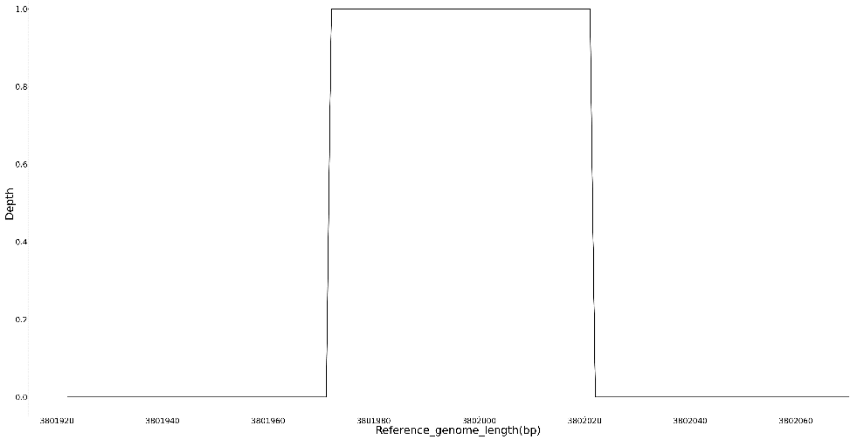

Yinxiaolin

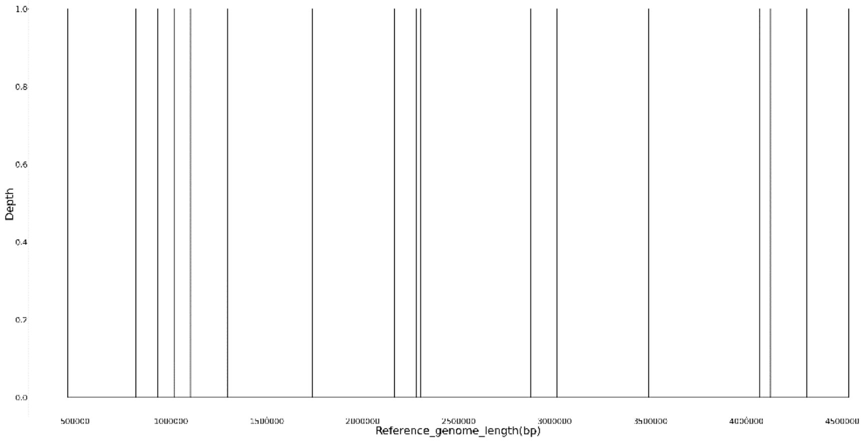

Zhanghaichen

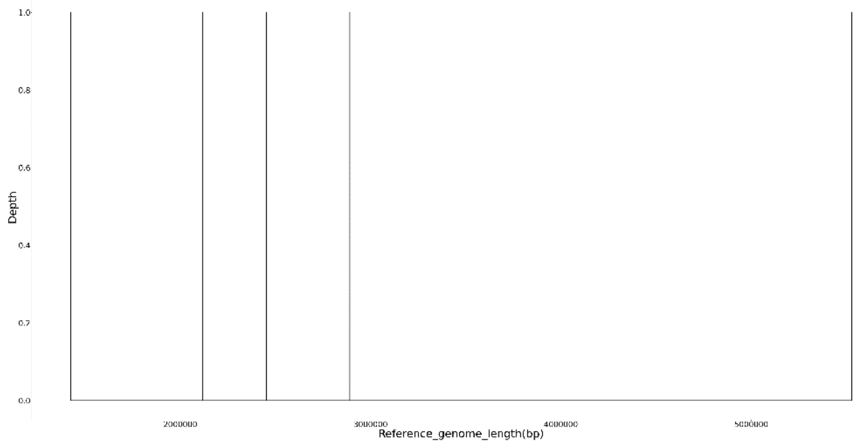

Zhanghailan

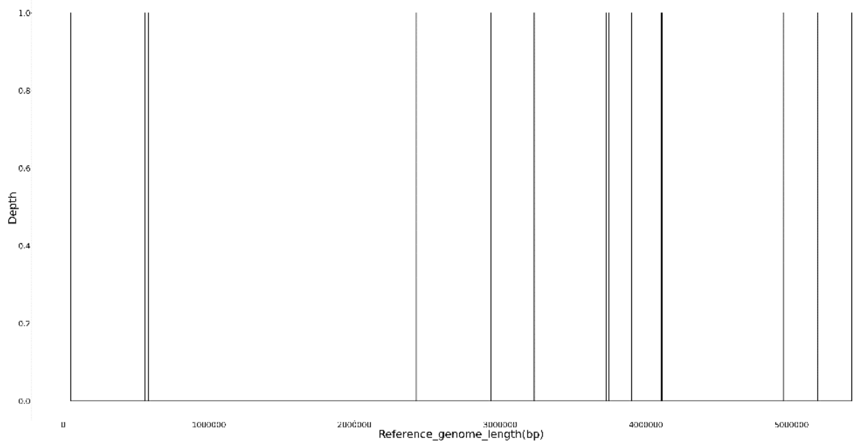

Zhangjie

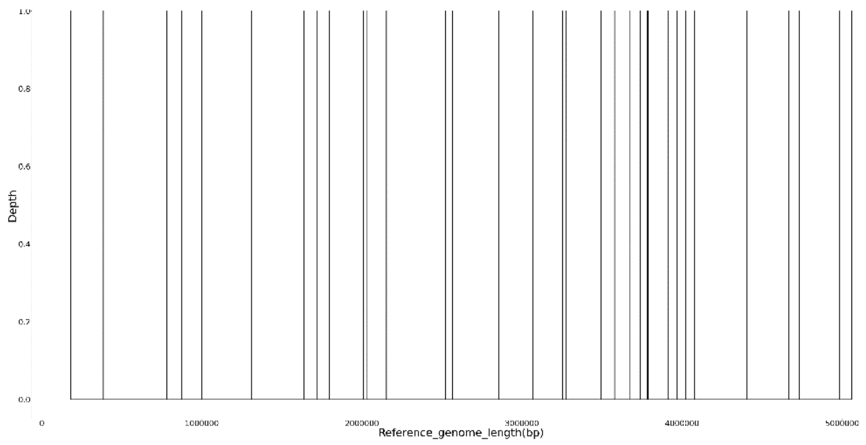

Zhangjiefang

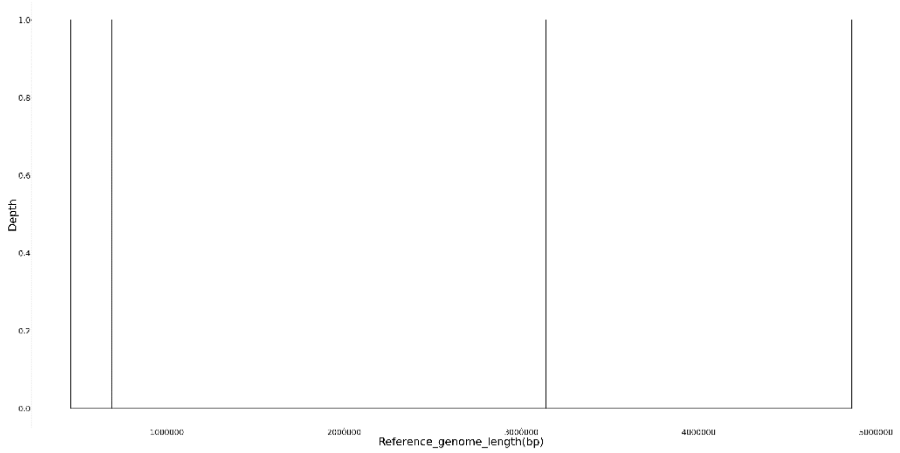

Zhangmaofan

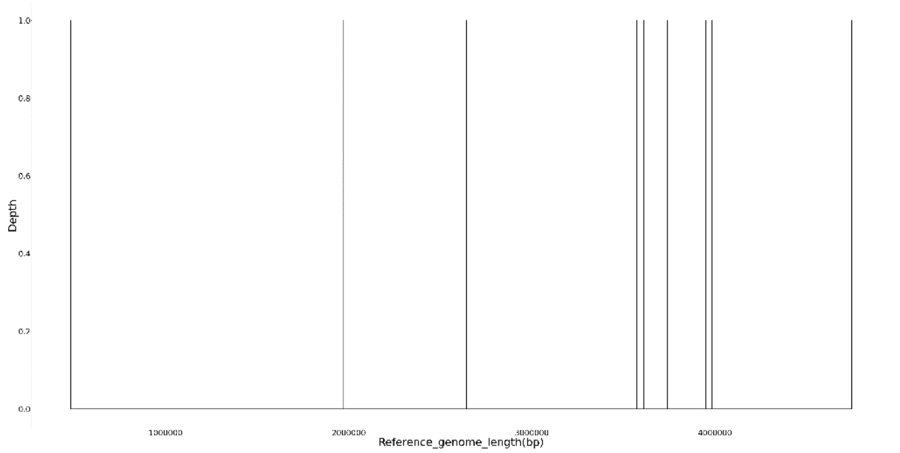

Zhangshifeng

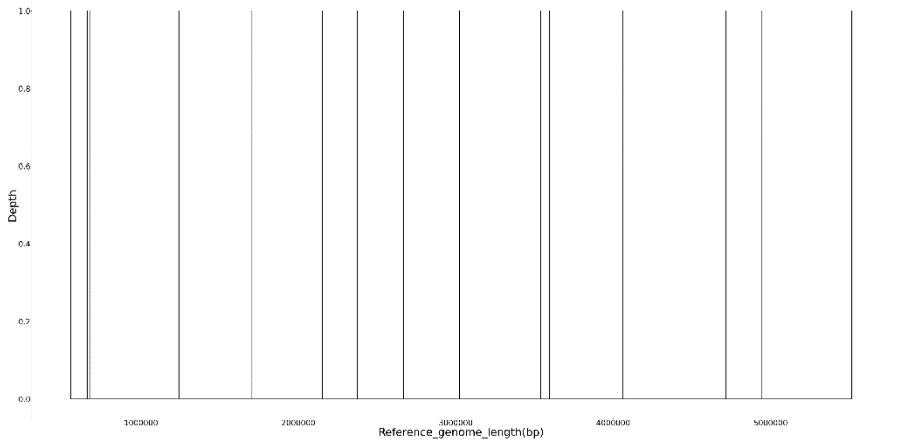

Zhangyana

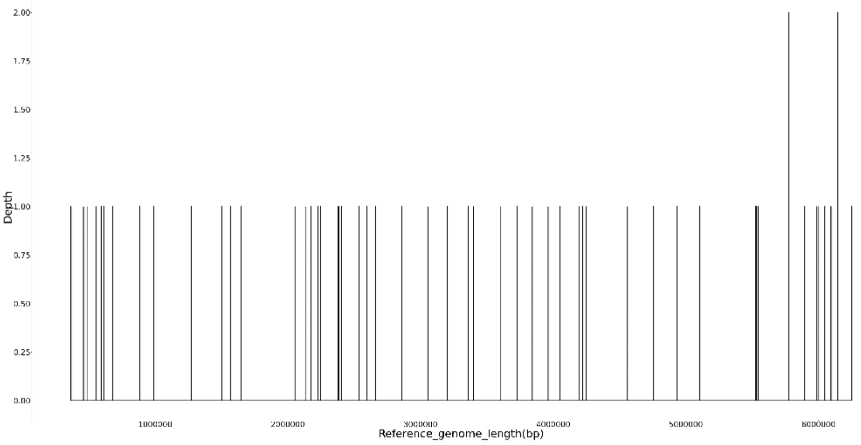

Zhaojianzhong

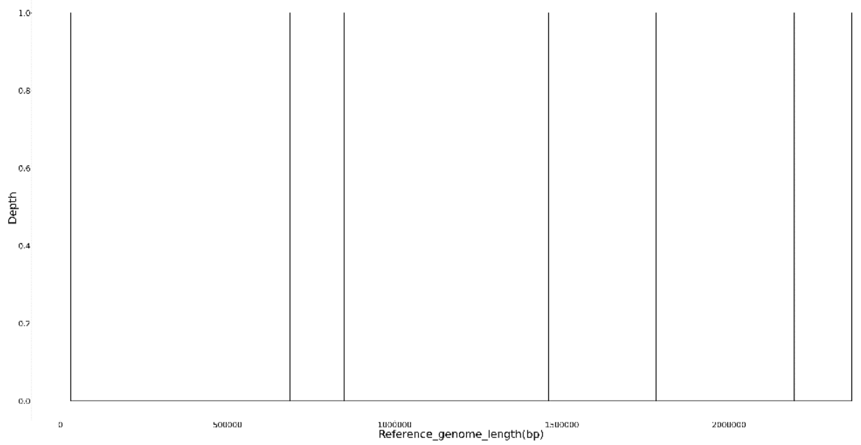

Zhoudiu

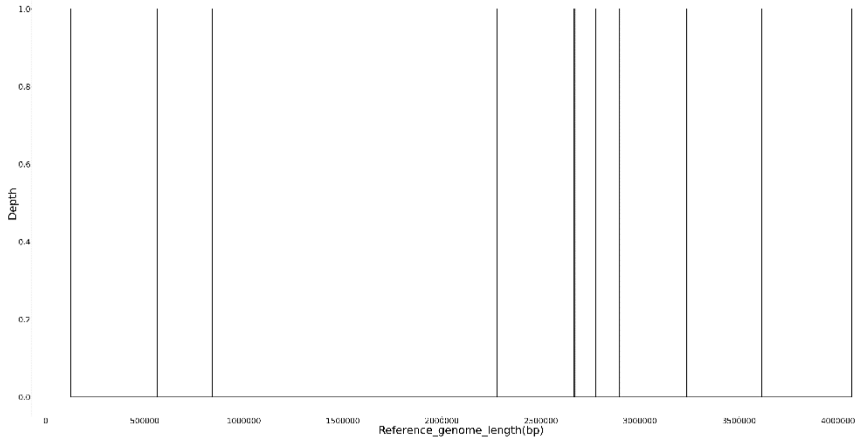

Supplement: Supplementary file 1 [file DataSheet_1.pdf]
